# The 7-phenyl benzoxaborole series is active against *Mycobacterium tuberculosis*

**DOI:** 10.1016/j.tube.2017.11.003

**Published:** 2018-01

**Authors:** Aaron Korkegian, Theresa O'Malley, Yi Xia, Yasheen Zhou, David S. Carter, Bjorn Sunde, Lindsay Flint, Dean Thompson, Thomas R. Ioerger, Jim Sacchettini, M.R.K. Alley, Tanya Parish

**Affiliations:** a TB Discovery Research, Infectious Disease Research Institute, Seattle, WA, USA; b Anacor Pharmaceuticals, Palo Alto, CA, USA; c Texas A&M University, College Station, TX, USA

## Abstract

We identified a series of novel 7-phenyl benzoxaborole compounds with activity against *Mycobacterium tuberculosis*. Compounds had a range of activity with inhibitory concentrations (IC_90_) as low as 5.1 μM and no cytotoxicity against eukaryotic cells (IC_50_ > 50 μM). Compounds were active against intracellular mycobacteria cultured in THP-1 macrophages. We isolated and characterized resistant mutants with mutations in NADH dehydrogenase (Ndh) or the regulatory protein Mce3R. Mutations suggest that Ndh may be the target of this series.

Tuberculosis (TB) represents a serious threat to global health as one of the top 10 causes of death worldwide with increasing rates of new infections [1]. Current treatments suffer from long treatment time and a growing problem of multidrug resistance. There is therefore a need for new molecules with anti-tubercular activity as well as new targets for the development of treatments.

The combination of two privileged structures [2], the biphenyls and the benzoxaboroles, identified a novel 7-phenyl benzoxaborole series with activity against *Mycobacterium tuberculosis in vitro*. Three compounds with a 7-phenyl benzoxaborole structure were tested for activity against *Mycobacterium tuberculosis* (Fig. 1 and Supplementary Information). Compounds had activity in both liquid and solid medium (Table 1). We determined inhibitory concentrations under aerobic conditions as previously described [3]. Compounds were solubilized in DMSO and tested as 10-point two-fold serial dilutions. Bacteria were cultured in Middlebrook 7H9 medium supplemented with 10% v/v OADC (oleic acid, albumin, dextrose, catalase) and 0.05% w/v Tween-80. Assay plates were inoculated with *M. tuberculosis* H37Rv LP (ATCC 25618) [4] and incubated for 5 days at 37 °C. Growth was measured using OD_590_ and plotted using the Levenberg-Marquart algorithm. The IC_90_ was defined as the concentration at which 90% growth was inhibited. All three compounds were active against replicating bacteria. AN11987 had good activity, with an IC_90_ of 5.1 μM (Table 1). AN6288 and AN6291 had less activity, with IC_90_ of 80 μM and 55 μM respectively (about 10-fold less active). Compounds were also active on solid medium. We determined the MIC_99_ (defined as the concentration required to prevent 99% growth) for each compound on Middlebrook 7H10 containing 10% v/v OADC. Again, AN11987 was the most active (MIC_99_ = 5 μM) as compared to AN6288 and AN6291 (MIC_99_ = 12.5 μM), although the difference was less pronounced (Table 1).

**Fig. 1:**
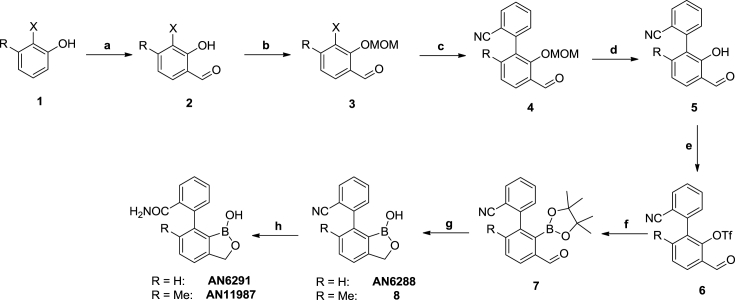
**Synthetic pathway of 7-phenyl benzoxaborole compounds**. R = H, X = Br; R = Me, X = I; a) (CH_2_O)_n_, MgCl_2_, TEA, MeCN, reflux, overnight, 90%; b) MOMCl, DIEA, DCM, 0 °C, 1 h, 90%; c) 2-cyanophenylboronic acid, Pd(PPh_3_)_2_Cl_2_, Na_2_CO_3_, 1,4-dioxane, H_2_O, 80 °C, 12 h, 46–56%; d) 2 N HCl (aq), THF, 50 °C, 4 h, 64–93%; e) Tf_2_O, pyridine, DMAP, DCM, rt, 2 h, 45–70%; f) (PinB)_2_, KOAc, Pd(PPh_3_)_2_Cl_2_, 1,4-dioxane, N_2_, 80 °C, 6 h, 67–87%; g) NaBH_4_, MeOH, THF, rt, 0.5 h; 6 N HCl (aq), rt, 0.5 h, 56–66%; h) NaOH, H_2_O, 80 °C, 8 h, 32–64%.

**Table 1:** **Biological activity of compounds.** Compounds were tested for activity in liquid or solid medium. MIC_99_ is the concentration required to inhibit growth by 99%. IC_50_ and IC_90_ are the concentrations required to reduce bacterial/cell viability in the intracellular macrophage activity assay by 50% and 90% respectively. Intracellular activity is against *M. tuberculosis*, cytotoxicity is against THP-1 cells.

Compound	Liquid	Solid	Intracellular activity		Cytotoxicity
	IC_90_ (μM)	MIC_99_ (μM)	IC_50_ (μM)	IC_90_ (μM)	IC_50_ (μM)
AN6288	80 ± 30 (n = 4)	12.5	15	30	>50
AN6291	55 ± 13 (n = 3)	12.5	3.9	5.0	>50
AN11987	5.1 ± 1.5 (n = 4)	5.0	0.69	1.8	>50

We also measured compound activity against intracellular bacteria. We infected the THP-1 human monocytic cell line (ATCC-TIB202). Briefly THP-1 cells were maintained in RPMI-1640, 10% v/v FBS, 2 mM Corning glutagro, 1 mM sodium pyruvate and differentiated using 80 nM phorbol 12-myristate 13-acetate. Cells were infected at a multiplicity of infection of 1 with *M. tuberculosis* constitutively expressing luciferase [5], washed to remove extra-cellular bacteria and seeded into 96-well plates containing compounds. Infected cells were cultured for 72 h and bacterial viability assessed by RLU. The cytotoxicity of compounds was determined against uninfected THP-1 cells exposed to compounds for 72 h; cell viability was measured using CellTiter-Glo (Promega). For both assays, curves were fitted using the Levenberg-Marquardt algorithm. The IC_50_ and IC_90_ were defined as the concentrations that reduced cell/bacterial viability by 50% or 90% respectively. All three compounds showed good intracellular activity with no cytotoxicity (IC_50_ > 50 μM) (Table 1). The activity trend seen in axenic culture was preserved against intracellular bacteria, with AN11987 being the most active, IC_90_ = 1.8 μM AN6288 was the least active, with IC_90_ = 30 μM. AN6291 had intermediate activity (IC_90_ = 5.0 μM).

Although the target for the 3-aminomethyl benzoxaboroles in *M. tuberculosis* is leucyl-tRNA synthetase (LeuRS) [6] the target and/or mechanism of resistance for these 7-phenyl benzoxaboroles compounds is unknown. We isolated resistant mutants on solid medium [7]. *M. tuberculosis* was plated onto solid medium containing 5X or 10X the MIC_99_. Colonies were isolated and confirmed for resistance in solid or liquid medium. We isolated and confirmed resistant mutants for AN6291 (7 mutants), AN6288 (2 mutants) and AN11987 (3 mutants). We selected three mutants resistant to AN6291 and two mutants resistant to AN6288 for whole genome sequencing.

Whole genome sequencing and mutation identification was conducting as described in Ioerger et al. 2010 [4] using an Illumina HiSeq 2500 and either 72 × 72bp paired-end reads or 54 × 54 paired-end reads. We identified a number of single nucleotide polymorphisms (SNPs) and a large deletion. Resistant mutants raised against AN6288 contained mutations within *ndh*, while resistant mutants raised against AN6291 contained mutations affecting *Mce3R* including an amino acid substitution, a stop codon and a large deletion (Table 2). Mutations were confirmed by PCR amplification and sequencing from genomic DNA. We sequenced *ndh* and *mce3R* in the remaining isolates. All the strains isolated against AN6291 had mutations in *mceR3*, whereas the strains isolated against AN6288 and AN11987 had mutations in *ndh* (Table 2).

**Table 2:** **Mutations identified in resistant isolates.** Strains were isolated on 5X or 10X MIC on solid medium and were confirmed as resistant by determining MICs. AN6291-RM1 had a large deletion encompassing *mce3R*. Mutations were identified by whole genome sequencing in five strains (indicated by an asterisk). Mutations in the remaining strains were identified by PCR amplification and sequencing of the gene.

Compound	Strain	Gene(s)	Mutation	Amino Acid change
AN6288	RM1*	*ndh*	667C > A	P223T
AN6288	RM2*	*ndh*	1262 T > C	F421S
AN6291	RM1*	Rv1948c-Rv1970	deletion	
AN6291	RM2	*mce3R*	334 T > C	W112R
AN6291	RM3	*mce3R*	+149–150 deletion	
AN6291	RM4	*mce3R*	334 T > C	W112R
AN6291	RM5*	*mce3R*	563G > T	C188F
AN6291	RM6*	*mce3R*	555C > A	Y185*
AN6291	RM7	*mce3R*	+649–936 deletion	
AN11987	RM1	*ndh*	994G > A	V332 M
AN11987	RM2	*ndh*	994G > A	V332 M
AN11987	RM3	*ndh*	998C > T	A333V

We tested selected strains for cross-resistance; MICs were determined on solid medium using a modified version of the MIC_99_, using 96-well plates and a smaller inoculum to conserve compound. Again, we saw the same trend of activity with AN11987 being most active (MIC = 0.2 μM) (Table 3). Cross-resistance to all three compounds was observed in strains containing either *ndh* or *mce3R* mutations.

**Table 3:** **Cross-resistance profile of resistant strains.** MICs were measured on solid medium in 96-well format. MICs were defined as the minimum concentration required to inhibit growth completely. *AN6291-RM1 had a large deletion encompassing *mce3R*.

Strain	Gene	Mutation	Amino acid change	MIC solid (μM)		
				AN6288	AN6291	AN11987
H37Rv				12.5	12.5	0.2
AN6288-RM1	*ndh*	667C > A	P223T	50	50	25
AN6288-RM2	*ndh*	1262 T > C	F421S	50	50	3.1
AN6291-RM1	*	Rv1948c-Rv1970 deletion		50	100	3.1
AN6291-RM5	*mce3R*	563G > T	C188F	50	100	3.1
AN6291-RM6	*mce3R*	555C > A	Y185*	50	100	3.1

Ndh is an essential oxidoreductase, which catalyzes electron transfer from NADH to menoquinone as part of the electron transport chain [8]. Ndh is the target of several small molecule series including the phenothiazines [9] and quinolinyl pyrimidines [10]. Mutations in Ndh found in clinical isolates have also been associated with resistance to isoniazid (R13C, V18A, T110A, A226E, R268H, G313R and N316K) [11], [12], [13]. The mutations we identified (P223T, F421S, V332 M and A333V) have not previously been reported. However, alignment of *M. tuberculosis* Ndh with *Staphylococcus aureus* (pdb 4XDB) and *Caldalkalibacillus thermarum* (pdb 4NWZ) structures revealed that V332 and A333 correspond to residues involved in the quinone binding pocket [14], [15]. V332 (T315 in *C. thermarum* and T318 in *S. aureus*) is involved in NADH binding while A333 is (A316 in *C. thermarum* and A319 in *S. aureus*) is part of the conserved AQxAxQ motif common amongst NDH-2 analogs. Given that these Ndh mutations conferred resistance to all three compounds, we propose that Ndh is likely the target of this compound series, and that mutations lead to loss of binding. Further work to express, purify and co-crystallise the protein would address this question.

Mce3R is a TetR transcriptional repressor that negatively regulates genes involved in lipid metabolism and redox reactions [16]. The Mce3R mutations were inactivating mutations, including premature stop codons, a frameshift and a large deletion covering *mce3R* (Table 2). This suggests Mce3R might control genes involved in resistance, since deletion of Mce3R activity would lead to upregulation of regulon members. Genes controlled by Mce3R include a putative epoxide hydrolase (Rv1938), a monooxygenase (Rv1936), a methyltransferase (Rv1498c) and a succinate-semialdehyde dehydrogenase (Rv0234c) as well as several synthetases and conserved hypotheticals of unknown function. Since MceR3 is a regulatory protein, we propose that a regulon member might be involved in compound degradation or metabolism. It is interesting to note that the addition of 6-methyl group to AN6291 yielded a more potent compound, while the mutations all mapped to *ndh* not *mce3R*, which suggest that this methyl group might interfere with this resistance mechanism.

In conclusion, we identified a new benzoxaborole series, the 7-phenyl benzoxaboroles, that had activity against *M. tuberculosis in vitro* and may target Ndh rather than LeuRS. The 7-phenyl benzoxaboroles are effective against *M. tuberculosis* in axenic culture and in macrophage culture, with no cytotoxicity. We characterized two routes to resistance, one of which is via gene regulation (Mce3R) and the other in Ndh. It is possible that Ndh represents the target of the 7-phenyl benzoxaborole compounds while Mce3R has some effect on the target or on the compounds through activity of a gene within its regulon. We propose that this is a series with potential for further development and target validation work.

We thank James Ahn and Yulia Ovechkina for technical assistance.

## Funding

This research was supported with funding from the Bill and Melinda Gates Foundation (OPP1024038) and by NIAID of the National Institutes of Health under award number R01AI099188. The content is solely the responsibility of the authors and does not necessarily represent the official views of the National Institutes of Health.

## Appendix A Supplementary data

mmc1 mmc1

mmc2 mmc2
