# Supplementary material for: The 7-phenyl benzoxaborole series is active against Mycobacterium tuberculosis
Source: Tuberculosis (Edinb). 2018 Jan;108:96–8. doi: 10.1016/j.tube.2017.11.003 (PMC5854369; doi:10.1016/j.tube.2017.11.003)
Supplement: mmc2 [file mmc2.docx]

**Synthesis of AN6288 and AN6291**

**3-bromo-2-hydroxybenzaldehyde (2, R = H, X = Br):** To a solution of 2-bromophenol (86 g, 0.5 mol) in CH_3_CN (2 L) were added (CH_2_O)_n_ (60 g, 2 mol), MgCl_2_ (87 g, 1 mol) and TEA (353.5 g, 3.5 mol) at room temperature under N_2_. The mixture was refluxed overnight. Then to it was added 2M HCl (aq) and it was extracted with EtOAc. The organic layer was concentrated to give the crude product (90 g, 90%). ^1^H NMR (400 MHz, CDCl_3_): δ 11.64 (s, 1H), 9.88 (s, 1H), 7.80 (d, J = 7.6 Hz, 1H), 7.57 (d, J = 7.6 Hz, 1H), 6.97 (t, J = 7.6 Hz, 1H).

**3-bromo-2-(methoxymethoxy)benzaldehyde (3, R = H, X = Br):** To a solution of 3-bromo-2-hydroxybenzaldehyde (5 g, 25 mmol) and DIEA (7 mL, 50 mmol) in DCM (100 mL) was added MOMCl (3.02 g, 37.5 mmol) slowly at 0°C for 1 hour. Water was added and the mixture was extracted by DCM three times. The organic layer was concentrated to afford a yellow solid (5.6 g, 90%). ^1^H NMR (400 MHz, CDCl_3_): δ 10.32 (s, 1H), 7.80 (m, 2H), 7.13 (t, 1H), 5.18 (s, 2H), 3.60 (s, 3H).

**3'-formyl-2'-(methoxymethoxy)-[1,1'-biphenyl]-2-carbonitrile (4, R = H):** A mixture of 3-bromo-2-(methoxymethoxy)benzaldehyde (12.2 g, 50 mmol), K_3_PO_4_ (21.2 g, 100 mmol), 2-cyanophenylboronic acid (14.7 g, 100 mmol) and Pd(dppf)Cl_2_ (2 g, catalyst) in 1,4-dioxane (500 mL) was stirred under N_2_ at 80°C for 12 hours. Water was added and the mixture was extracted by ethyl acetate three times. The organic layer was concentrated to afford a yellow solid (6.2 g, 46%). ^1^H NMR (400 MHz, CDCl_3_): δ 10.42 (s, 1 H), 7.95 (m, 2 H), 7.56 (m, 4 H), 7.38 (m, 1 H), 4.72 (s, 2 H), 3.12 (s, 3H).

**3'-formyl-2'-hydroxy-[1,1'-biphenyl]-2-carbonitrile (5, R = H):** 3'-formyl-2'-(methoxymethoxy)-[1,1'-biphenyl]-2-carbonitrile (12 g, 44.94 mmol) was dissolved in THF (500 mL), and then 2M HCl (250 mL) was added into the reaction mixture. The solution was stirred at 50°C for 4 hours. The reaction mixture was extracted by DCM three times. The organic layer was concentrated to afford a yellow solid (7 g, yield 64%). ^1^H NMR (400 MHz, CDCl_3_): δ 11.51 (s, 1 H), 10.05 (s, 1 H), 7.70 (m, 6 H), 7.13 (t, 1 H).

**2'-cyano-3-formyl-[1,1'-biphenyl]-2-yl trifluoromethanesulfonate (6, R = H):** 3'-formyl-2'-hydroxy-[1,1'-biphenyl]-2-carbonitrile (5 g, 22.42 mmol), pyridine (17.5 g, 224.2 mmol) and DMAP (100 mg, catalyst) in DCM (300 mL) was added Tf_2_O (12.5 g, 44.84 mmol) slowly at 0^o^C for 10 mins. The reaction mixture was stirred at rt for 2 hours. Water was added and the mixture was extracted by DCM three times. The organic layer was concentrated and purified by column to afford a yellow solid (5.5 g, yield 70%).

**3'-formyl-2'-(4,4,5,5-tetramethyl-1,3,2-dioxaborolan-2-yl)-[1,1'-biphenyl]-2-carbonitrile (7, R = H):** 2'-cyano-3-formyl-[1,1'-biphenyl]-2-yl trifluoromethanesulfonate (5.5 g, 15.5 mmol), KOAc (3.04 g, 31 mmol), 4,4,4',4',5,5,5',5'-octamethyl-2,2'-bi(1,3,2-dioxaborolane) (7.87 g, 31 mmol), and Pd(dppf)Cl_2_ (1 g, catalyst) was added in 1,4-dioxane (200 mL). Then the reaction mixture was stirred under N_2_ at 80°C for 6 hours. Water was added and the mixture was extracted by ethyl acetate three times. The organic layer was concentrated and purified by column to afford a brown solid (4.5 g, yield 87%).

**2-(1-hydroxy-1,3-dihydrobenzo[c][1,2]oxaborol-7-yl)benzonitrile (AN6288):** To a solution of 3'-formyl-2'-(4,4,5,5-tetramethyl-1,3,2-dioxaborolan-2-yl)-[1,1'-biphenyl]-2-carbonitrile (6 g, 18.01 mmol) in a mixed solvent of MeOH (10 mL) and THF (100 mL) was added NaBH_4_ (1.37 g, 36 mmol). The reaction mixture was stirred at rt for 30 mins. Then the mixture was adjusted to pH = 2-3 with 12 N HCl and stirred for 30 mins. The solvent was removed and the residue was added water and EtOAc. The organic layer was washed with brine, dried over Na_2_SO_4_ and concentrated to give the crude product. [Recrystallization](app:ds:recrystallization) with EtOAC/petroleum ether (1:5, v/v) to give **AN6288** (2.8 g, 66%). ^1^H NMR (400 MHz, CDCl_3_): δ 8.77 (s, 1 H), 7.82 (m, 1 H), 7.57 (m, 5 H), 7.33 (m, 1 H), 5.06 (s, 2H).

**2-(1-hydroxy-1,3-dihydrobenzo[c][1,2]oxaborol-7-yl)benzamide (AN6291):** To a mixture of 2-(1-hydroxy-1,3-dihydrobenzo[c][1,2]oxaborol-7-yl)benzonitrile (360 mg, 1.53 mmol) in water (15 mL) was added NaOH (360 mg, 9 mmol). The mixture was heated to 80°C for 8 hrs. The mixture was concentrated *in vacuo* and the residue was purified by preparative HPLC providing **AN6291** (0.25 g, 64%).

**Synthesis of AN11987**

**2-iodo-3-methylphenol (1, R = Me, X = I):** A solution of NaNO_2_ (7.47 g, 108 mmol) in water (100 mL) was added dropwise to a solution of 2-amino-3-methylphenol (15 g, 100 mmol) in 1 N HCl (200 mL) at 0°C. After addition was completed, the mixture was stirred for additional 30 mins and then slowly added to a solution of KI (24.4 g, 147 mmol) in water (180 mL) at 0°C. After addition was completed, the mixture was warmed to r.t. and stirred overnight. The mixture was neutralized with solid KOH and extracted with EtOAc. The organic layer was washed with brine, dried over anhydrous Na_2_SO_4_, and concentrated to afford a light yellow solid (24.1 g, 72%).

**2-hydroxy-3-iodo-4-methylbenzaldehyde (2, R = Me, X = I ):** To solution of 2-iodo-3-methylphenol (86 g, 0.5 mol) in CH_3_CN (2 L) was added (CH_2_O)_n_ (60 g, 2 mol), MgCl_2_ (87 g, 1 mol) and TEA (353.5 g, 3.5 mol) at room temperature under N_2_. The mixture was stirred at reflux overnight. Then 2M HCl (aq) was added and the mixture was extracted with EtOAc. The organic layer was concentrated to give the crude product (90 g, 90%).

**3-iodo-2-(methoxymethoxy)-4-methylbenzaldehyde (3, R = Me, X = I):** To a solution of 2-hydroxy-3-iodo-4-methylbenzaldehyde (5 g, 25 mmol) and DIEA (7 mL, 50 mmol) in DCM (100 mL) was added MOMCl (3.02 g, 37.5 mmol) slowly at 0°C for 1 hour. Water was added and the mixture was extracted by DCM three times. The organic layer was concentrated to afford a yellow solid (5.6 g, 90%).

**3'-formyl-2'-(methoxymethoxy)-6'-methyl-[1,1'-biphenyl]-2-carbonitrile (4, R = Me):** 3-iodo-2-(methoxymethoxy)-4-methylbenzaldehyde (2 g, 6.5 mmol), Na_2_CO_3_ (2.07 g, 19.5 mmol), 2-cyanophenylboronic acid (2.98 g, 13 mmol) and Pd(PPh_3_)_2_Cl_2_ (0.05 g, catalyst) was added in 1,4-dioxane/H_2_O (15 mL:1 mL). Then the reaction mixture was stirred under N_2_ at 80°C for 12 hours. Water was added and the mixture was extracted by ethyl acetate three times. The organic layer was concentrated and the residue was purified by column to afford a yellow solid (1 g, 56%).

**3'-formyl-2'-hydroxy-6'-methyl-[1,1'-biphenyl]-2-carbonitrile (5, R = Me):** 3'-formyl-2'-(methoxymethoxy)-6'-methyl-[1,1'-biphenyl]-2-carbonitrile (1 g, 3.5 mmol) was dissolved in THF (10 mL), and then 2M HCl (10 mL) was added into the reaction mixture. The solution was stirred at 40°C for 2 hours. The reaction mixture was extracted by DCM three times. The organic layer was concentrated to afford a yellow solid (1.0 g, 93%), which was used directly for the next step.

**2'-cyano-3-formyl-6-methyl-[1,1'-biphenyl]-2-yl trifluoromethanesulfonate (6, R = Me):** To a mixture of 3'-formyl-2'-hydroxy-6'-methyl-[1,1'-biphenyl]-2-carbonitrile (0.7 g, 2.9 mmol), pyridine (1 mL), and DMAP (10 mg, catalyst) in DCM (10 mL) was added Tf_2_O (1.65 g, 5.8 mmol) slowly at 0°C for 10 mins. The reaction mixture was stirred at 0°C for 2 hours. Water was added and the mixture was extracted by DCM three times. The organic layer was concentrated and purified by column to afford a yellow solid (0.5 g, 45%).

**3'-formyl-6'-methyl-2'-(4,4,5,5-tetramethyl-1,3,2-dioxaborolan-2-yl)-[1,1'-biphenyl]-2-carbonitrile (7, R = Me):** A mixture of 2'-cyano-3-formyl-6-methyl-[1,1'-biphenyl]-2-yl trifluoromethanesulfonate (0.5 g, 1.3 mmol), KOAc (0.25 g, 2.6 mmol), (PinB)_2_ (0.66 g, 2.6 mmol) and Pd(PPh_3_)_2_Cl_2_ (0.09 g, catalyst) in 1,4-dioxane (10 mL) was stirred under N_2_ at 80°C overnight. Water was added and the mixture was extracted by ethyl acetate three times. The organic layer was concentrated and the residue was purified by column to afford a brown solid (0.3g, 67%).

**2-(1-hydroxy-6-methyl-1,3-dihydrobenzo[c][1,2]oxaborol-7-yl)benzonitrile (8, R = Me):** To a solution of 2'-cyano-3-formyl-6-methyl-[1,1'-biphenyl]-2-yl trifluoromethanesulfonate (300 mg, 0.86 mmol) in MeOH (0.5 mL) and THF (5 mL) was added NaBH_4_ (65 mg, 1.7 mmol) and the mixture was stirred for 1 hour at r.t. Then the mixture was adjusted to pH = 2-3 with 6 N HCl and stirred for 30 mins. The solvent was removed and the residue was added to water and EtOAc. The organic layer was washed with brine, dried over Na_2_SO_4_, and concentrated to give the crude product, which was purified by preparativeHPLC to obtained pure compound **8** (120 mg, 56%). ^1^H NMR (400 MHz, CDCl_3_): δ 8.52 (s, 1H), 7.90-7.88 (m, 1H), 7.73-7.72 (m, 1H), 7.49-7.47 (m, 1H), 7.42-7.41 (m, 1H), 7.40-7.39 (m, 1H), 5.02 (s, 2H), 2.01 (s, 3H).

**2-(1-hydroxy-6-methyl-1,3-dihydrobenzo[c][1,2]oxaborol-7-yl)benzamide (AN11987):** 2-(1-hydroxy-6-methyl-1,3-dihydrobenzo[c][1,2]oxaborol-7-yl)benzonitrile (100 mg, 0.4 mmol) was added to a solution of NaOH (100 mg, 2.5 mmol) in water (8 mL) at room temperature, and then the mixture was heated to 100^o^C and stirred for 8 hrs. The mixture was then cooled to 0°C and HCl (2N) was added until pH=4. white solid was precipitated, and the mixture was filtered to give crude product, which was purified by preparativeHPLC (column: Phenomenex luna C18 250*80 mm*10 µm; mobile phase: [water (0.1%TFA)-ACN]; B%: 25%-55%, 20 min) to give **AN11987** (32 mg, yield 32%) as white solid. ^1^H NMR (400 MHz, DMSO): δ 8.24 (s, 1H), 7.59-7.57 (m, 1H), 7.44-7.40 (m, 2H), 7.32-7.30 (m, 1H), 7.25-7.23 (m, 1H), 7.07-7.05 (m, 2H), 6.81 (s, 1H), 4.96 (s, 2H), 2.01 (s, 3H).
